# Grape Composition under Abiotic Constrains: Water Stress and Salinity

**DOI:** 10.3389/fpls.2017.00851

**Published:** 2017-05-30

**Authors:** José M. Mirás-Avalos, Diego S. Intrigliolo

**Affiliations:** Departamento de Riego, Centro de Edafología y Biología Aplicada del Segura, Consejo Superior de Investigaciones CientíficasMurcia, Spain

**Keywords:** anthocyanins, deficit irrigation, saline water, sugars, titratable acidity, *Vitis vinifera* L., wine

## Abstract

Water stress and increasing soil salt concentration represent the most common abiotic constrains that exert a negative impact on Mediterranean vineyards performance. However, several studies have proven that deficit irrigation strategies are able to improve grape composition. In contrast, irrigation with saline waters negatively affected yield and grape composition, although the magnitude of these effects depended on the cultivar, rootstock, phenological stage when water was applied, as well as on the salt concentration in the irrigation water. In this context, agronomic practices that minimize these effects on berry composition and, consequently, on wine quality must be achieved. In this paper, we briefly reviewed the main findings obtained regarding the effects of deficit irrigation strategies, as well as irrigation with saline water, on the berry composition of both red and white cultivars, as well as on the final wine. A meta-analysis was performed using published data for red and white varieties; a general liner model accounting for the effects of cultivar, rootstock, and midday stem water potential was able to explain up to 90% of the variability in the dataset, depending on the selected variable. In both red and white cultivars, berry weight, must titratable acidity and pH were fairly well simulated, whereas the goodness-of-fit for wine attributes was better for white cultivars.

## Introduction

Grape quality is a complex concept that mainly refers to berry chemical composition, including sugars, acids, phenolics, and other aroma compounds (Lund and Bohlmann, 2006). The composition and concentration of these chemical compounds change during berry development and can be affected by many factors, either environmental, endogenous, or management practices (Jackson and Lombard, 1993; Dai et al., 2011). In this context, climate change will pose relevant constraints to grape and wine production in the coming years (Santos et al., 2012). Increasing temperatures, lower rainfall amounts, and heat waves are expected to become more frequent over the course of this century (IPCC, 2014). However, the most imminent challenges that grape, wine, and raisin industries must face, especially in arid and semi-arid regions, are increasing drought and salinity due to higher evaporation and declining water availability (Schultz and Stoll, 2010).

The effects of water stress on grapevine (*Vitis vinifera* L.) metabolism, vegetative development, productive performance, and berry composition have been widely studied for many combinations of rootstocks, cultivars, and climate conditions (e.g., Acevedo-Opazo et al., 2010; Intrigliolo et al., 2016). However, the extents to which berry secondary metabolites and wine composition are affected by water stress have seldom been assessed.

Salinity effects on vine performance and berry composition have been studied mainly in Australia (e.g., Stevens et al., 2011; Walker et al., 2014) but research in other areas is scarce. Reported results suggest that cultivar, rootstock, salt concentration, and time of exposure to saline conditions are relevant factors for the final berry and wine composition.

This review summarizes the main findings on the effects of water and salinity stresses on berry and wine composition, both in red and white cultivars.

## Water Stress

Water is critical for viticulture sustainability because grape production, quality, and economic viability largely depend on water availability (Medrano et al., 2015). A great effort has been devoted to assess the influence of grapevine water status on berry composition, mainly on red varieties under semi-arid conditions, accounting for total soluble solids (TSS), titratable acidity, and pH, although some other traits such as malic and tartaric acid concentrations, phenolics, anthocyanins, and tannins have been considered in some studies (e.g., Peyrot des Gachons et al., 2005; Bindon et al., 2008; van Leeuwen et al., 2009). However, detailed assessments of aroma precursors (Savoi et al., 2016), individual anthocyanins (Bindon et al., 2008, 2011; Santesteban et al., 2011; Cook et al., 2015; Hochberg et al., 2015), or phenolics (Ojeda et al., 2002; Ollé et al., 2011) have rarely been undertaken.

Reported results suggest that many factors including genotypes, climate, soil, and vineyard management can influence vine response to water stress, as reviewed by Medrano et al. (2015) and confirmed by the meta-analysis reported by Lavoie-Lamoureux et al. (2017). In water-limited areas, deficit irrigation practices can be a useful tool for manipulating berry composition to enhance and modulate the season-to-season variability in red wine composition (Intrigliolo et al., 2012), leading to changes in wine sensory properties (Chapman et al., 2005). However, the intensity of water stress and its period of occurrence over the grapevine growing cycle are of paramount importance. Apart from the variability in the response due to genotypes, environment, experimental setup, management practices among others, water stress imposed at pre-veraison stages induces major metabolic modifications in the berry that can be maintained even after re-watering (Shellie, 2014; Keller et al., 2016). In contrast, post-veraison water deficit effects are more variable, preventing a generalization of its positive or negative influences (Girona et al., 2009; Intrigliolo and Castel, 2010; Munitz et al., 2017).

In general, a moderate water stress reduces berry weight and titratable acidity but increases TSS, total anthocyanins, and phenolics concentrations in red grapes (Romero et al., 2010), improving berry quality. However, when a certain threshold of water stress is surpassed, these beneficial effects are no longer observed. This response seems to depend on the combination rootstock/cultivar as well as on soil and climate conditions. Water potential is the main indicator of vine water status and some authors established relations between this indicator and berry compositional traits (Salón et al., 2005; van Leeuwen et al., 2009; Romero et al., 2010; Shellie and Bowen, 2014); however, these relationships differ amongst cultivars, region, year, soil types, and management practices. Usually, higher levels of water stress are reported to reduce berry weight and malic acid concentrations while increasing anthocyanins and sugar contents up to a threshold were they are negatively affected. However, these responses depend on other factors such as crop load, vineyard age, fertilization, soil type, berry maturation stage at harvest, and canopy development, amongst others. Furthermore, few studies have accounted for the effects that water stress might exert on berry skin and seeds (Ojeda et al., 2002; Roby and Matthews, 2004; Bucchetti et al., 2011; Merli et al., 2015), even though this issue is relevant to discern between the effects of dilution of components or to what extent water stress is affecting compound synthesis and metabolism. Ojeda et al. (2002) imposed three levels of water deficit to Shiraz grapevines and observed that the concentration of phenolic compounds increased in berry skins due to berry size reduction; however, timing of stress occurrence and its severity could lead to negative effects on phenolic compound concentrations. Ollé et al. (2011) observed that water deficits affected differently the anthocyanin composition of Shiraz berries and suggested a differential regulation of the genes involved in the last steps of the anthocyanin biosynthesis pathway. In this sense, Hochberg et al. (2015) found that water stress modified polyphenol metabolism of Shiraz and Cabernet Sauvignon depending on the phenological stage, inducing the accumulation of stress-related metabolites such as proline and ascorbate. Cook et al. (2015) reported that sustained deficit irrigation increased the concentrations of di-hydroxylated anthocyanins while regulated deficit irrigation increased those of tri-hydroxylated anthocyanins. It has been shown that grapevine responds to drought by modulating several secondary metabolic pathways, altering the abundance of some transcripts and metabolites involved in phenyl propanoid, isoprenoid, carotenoid, amino acid, and fatty acid metabolism, as observed for Cabernet Sauvignon and Chardonnay (Deluc et al., 2009) and Sauvignon vert (Savoi et al., 2016). This might affect flavor and quality characteristics of grapes and wines.

In this context, we attempted a meta-analysis using published data from irrigation studies in field-grown red and white grapevine varieties from several wine regions worldwide (Supplementary Table 1). A search under the terms “grapevine,” “water stress,” and “berry composition” was carried out in the Web of Knowledge database. This yielded 184 references. Those works referred to potted vines and those not including leaf (Ψ_l_) or stem (Ψ_stem_) water potential measurements were discarded. When only one of these measurements was present, we used the relationships reported by Intrigliolo and Castel (2006) to obtain the values for the missing one. In the end, 48 works have been used (Supplementary Table 1).

Data retrieval from publications was carried out similarly to that described in Lavoie-Lamoureux et al. (2017). The following information was associated to the data in the database, when available: cultivar, country, year, treatment, rootstock, and developmental stage in which water stress was imposed (pre- or post-veraison).

Data were analyzed using the IBM SPSS Statistics for Windows, version 23.0 (IBM Corp., Armonk, NY, United States). Data on berry size and composition (and wine attributes, when available) were used as dependent variables, while Ψ_stem_ was considered as a covariable and cultivar, rootstock, and timing when water stress was imposed as fixed factors. Moreover, Pearson’s coefficient of correlation was used to assess the relationships among vine water status (as determined by predawn, leaf, and stem water potentials) and berry size and composition.

The final database contained 420 data points (298 for red and 122 for white varieties) obtained from 48 references published between 1979 and 2017 (Supplementary Table 1). Twenty different *Vitis vinifera* cultivars were represented (11 red and 9 white). The number of data retrieved per publication varied between 2 and 28, averaging 10.5. Merlot and Tempranillo were the most represented varieties (79 and 78 data, respectively) among the red ones. In the case of white cultivars, Sauvignon blanc showed the highest number of data with 48.

For red varieties, cultivar, timing, and Ψ_stem_ intervened significantly on the model; whereas for white varieties the factors were cultivar, rootstock, and Ψ_stem_ (**Table 1**). No significant interactions among factors were detected. Overall, the models explained between 2 and 99.4% of the variation in the data distribution, depending on the variable considered (Supplementary Table 2).

**Table 1:** Factors included in the first univariate general linear model performed on the red and white varieties databases.

Factors	*p*-value
**Red varieties**	
Cultivar	<0.001
Rootstock	ns
Timing	<0.05
Ψ_stem_	<0.05
Cultivar × rootstock	ns
Cultivar × timing	ns
Cultivar × Ψ_stem_	ns
Roostock × timing	ns
Rootstock × Ψ_stem_	ns
Timing × Ψ_stem_	ns
Cultivar × rootstock × timing	ns
Cultivar × rootstock × Ψ_stem_	ns
Cultivar × timing × Ψ_stem_	ns
Rootstock × timing × Ψ_stem_	ns
Cultivar × rootstock × timing × Ψ_stem_	ns
**White varieties**	
Cultivar	<0.001
Rootstock	<0.01
Timing	ns
Ψ_stem_	<0.01
Cultivar × rootstock	ns
Cultivar × timing	ns
Cultivar × Ψ_stem_	ns
Roostock × timing	ns
Rootstock × Ψ_stem_	ns
Timing × Ψ_stem_	ns
Cultivar × rootstock × timing	ns
Cultivar × rootstock × Ψ_stem_	ns
Cultivar × timing × Ψ_stem_	ns
Rootstock × timing × Ψ_stem_	ns
Cultivar × rootstock × timing × Ψ_stem_	ns

*Non-significant factors were not included in the final model. ns, non-significant.*

The different measurements for assessing vine water status (predawn, leaf, and stem water potentials) considered in the current study were significantly related to several grape and wine compositional attributes. In the case of red varieties, midday stem water potential measured before veraison (Ψ_stempre_) was significantly and positively correlated to berry weight and must titratable acidity, whereas it was negatively correlated to must TSS, malic acid concentration and wine pH, tartaric acid, anthocyanins, and total phenolic index (TPI) (**Table 2**). The other measurements of water status were significantly correlated with a lower number of attributes; for instance, Ψ_stem_ was positively correlated with berry weight and must titratable acidity and negatively correlated to TSS (**Table 2**). However, Ψ_stem_ is the most widely used measurement for assessing vine water status and thus we used it for depicting the relationships between berry traits and water stress.

**Table 2:** Pearson’s correlation coefficients (*r*) among different modalities of vine water status assessment and berry size and compositional traits for red cultivars.

		Ψ_pd_	Ψ_stempre_	Ψ_stempost_	Ψ_stem_
Berry weight	*r*	**0.284**	**0.567**	**0.326**	**0.406**
	Significance	**0.000**	**0.000**	**0.000**	**0.000**
	*n*	**255**	**107**	**119**	**255**
Total soluble solids	*r*	**-0.199**	**-0.419**	**-0.453**	**-0.234**
	Significance	**0.001**	**0.000**	**0.000**	**0.000**
	*n*	**286**	**126**	**138**	**286**
pH	*r*	**-0.161**	–0.218	0.002	–0.090
	Significance	**0.024**	0.055	0.989	0.208
	*n*	**196**	78	90	196
Titratable acidity	*r*	**0.171**	**0.200**	0.166	**0.162**
	Significance	**0.005**	**0.040**	0.072	**0.008**
	*n*	**266**	**106**	118	**266**
Malic acid	*r*	0.065	**0.384**	0.137	0.086
	Significance	0.536	**0.040**	0.479	0.411
	*n*	94	**29**	29	94
Tartaric acid	*r*	0.052	–0.128	0.187	0.082
	Significance	0.641	0.507	0.332	0.458
	*n*	84	29	29	84
Anthocyanins	*r*	–0.029	–0.027	0.413	–0.021
	Significance	0.796	0.914	0.079	0.852
	*n*	81	19	19	81
Total phenolics index	*r*	0.145	–0.276	0.254	0.140
	Significance	0.269	0.173	0.211	0.285
	*n*	60	26	26	60
Wine alcohol	*r*	–0.213	–0.140	–0.273	–0.189
	Significance	0.055	0.402	0.097	0.088
	*n*	82	38	38	82
Wine titratable acidity	*r*	0.168	0.313	0.172	0.187
	Significance	0.164	0.081	0.347	0.121
	*n*	70	32	32	70
Wine pH	*r*	0.003	**-0.680**	0.229	–0.101
	Significance	0.977	**0.000**	0.208	0.408
	*n*	70	**32**	32	70
Wine malic acid	*r*	–0.004	–0.144	–0.128	–0.046
	Significance	0.973	0.430	0.485	0.707
	*n*	70	32	32	70
Wine tartaric acid	*r*	0.050	**-0.619**	–0.156	–0.126
	Significance	0.720	**0.001**	0.445	0.364
	*n*	54	**26**	26	54
Wine anthocyanins	*r*	0.049	**-0.434**	–0.047	0.046
	Significance	0.676	**0.013**	0.800	0.693
	*n*	76	**32**	32	76
Wine total phenolics index	*r*	–0.061	**-0.623**	–0.265	–0.068
	Significance	0.604	**0.000**	0.143	0.562
	*n*	76	**32**	32	76

*Significant correlations are shown in bold. Ψ_*pd*_, pre-dawn leaf water potential; Ψ_*stempre*_, pre-veraison midday stem water potential; Ψ_*stempost*_, post-veraison midday stem water potential; Ψ_*stem*_, midday stem water potential. Significance indicates the *p*-value for each correlation. n, number of data points.*

In the case of red varieties, berry weight tended to decline with increasing Ψ_stem_; however, the slope of this decrease depended on the cultivar (Supplementary Figure 1). This suggests that genetics may play a relevant role on the response of grapevines to water stress and those cultivars with small berries, such as Cabernet Sauvignon, suffer less important reductions when they are grown within a given interval of Ψ_stem_. In contrast, cultivars with large berries, such as Bobal or Merlot, seem to be more sensitive to little variations in grapevine water status.

The dataset did not show a clear relation between Ψ_stem_ and TSS in the berries (Supplementary Figure 1). Nevertheless, a certain degree of water stress (up to -1.3 MPa) was beneficial for sugar accumulation in the berry. When this threshold was surpassed, the concentration of TSS decreased. Similarly, no clear trend was observed for titratable acidity and anthocyanins (Supplementary Figure 1). However, Merlot berries showed a steep increase in the concentration of anthocyanins when Ψ_stem_ varied from -0.5 to -1.5 MPa.

Winemaking was not usually involved in the experimental design and a low amount of data was available for performing this meta-analysis. The lack of significant relations may have been caused by the variability in winemaking procedures in the different studies (yeast strain, fermentation temperature, and time, etc.). However, lower pre-veraison midday stem water potentials were significantly correlated with higher values of anthocyanins and TPI in wines (**Table 2**).

In the case of white varieties, data availability is much lower and, usually, studies are referred to cool climates. In this case, a significant but slight trend to lower berry weights with increasing water stress was observed (Supplementary Table 3). Cultivar seems to have a strong effect since Albariño berries remained almost unaffected whereas Sauvignon Blanc or Riesling berries were strongly reduced in terms of weight when Ψ_stem_ became more negative (Supplementary Figure 2). It must be noticed, however, that the levels of water stress experienced by the different cultivars were not the same.

In white cultivars, TSS seemed to be unaffected by water stress when the whole dataset was accounted for (Supplementary Figure 2). However, Riesling, Godello, Albariño, or Treixadura tended to show high TSS values with increasing water stress. In contrast, Muscat and Sauvignon Blanc showed similar TSS for various levels of Ψ_stem_. Although slight, a significant reduction in titratable acidity with increasing water stress was detected (Supplementary Figure 2). This relation was more marked in Godello and Riesling. Since these data come from experiments performed in cool climates, severe water restriction has rarely been achieved and Ψ_stem_ varied within narrow ranges. Only for Sauvignon Blanc and Muscat, Ψ_stem_ reached values close to -1.5 MPa or even more negative. Similarly to red cultivars, winemaking has rarely been carried out. Nevertheless, a trend to higher alcohol contents with increasing Ψ_stem_ was observed (data not shown). In addition, more negative Ψ_stem_ values led to lower titratable acidities (Supplementary Figure 2).

Water stress or deficit irrigation strategies effects on wine volatiles have seldom been assessed; likely because winemaking practices can modulate wine composition to a great extent (Ilc et al., 2016). Recently, Talaverano et al. (2017) reported that higher alcohols such as 2-methyl-1-butanol and 2,3-butanediol, as well as C6 compounds such as 1-hexanol increased in Tempranillo wines under water stress conditions in Western Spain; in contrast, 2-phenylethyl acetate concentration was significantly decreased by water deficit. Despite the fact that other 16 compounds did not present significant differences caused by vine water status, changes in 2-phenylethyl acetate might have consequences on wine sensory perception because this compound provides floral and sweet notes, whereas 2-methyl-1-butanol, 2,3-butanediol, and 1-hexanol provide malt, burned and creamy notes to wines. In this line of work, Mendez-Costabel et al. (2014) reported that moderate water stress would reduce 3-isobutyl-2-methoxypyrazine concentration, and thus the intensity of green aromas, without altering that of C6 compounds in Merlot grapes and wines. Finally, Ou et al. (2010) observed that deficit irrigation affected the concentrations of terpene alcohols and norisoprenoids in wines, whereas it had not consistent influence on ester concentrations.

## Saline Stress

Continued rates of water extraction for agriculture, declining rainfall trends and increased portioning of water for ecosystem servicing have led to unsustainable levels of water consumption in many parts of the world (Hamilton et al., 2007). This has focused the attention on the use of alternative water sources such as municipal and winery wastewaters for irrigation instead of scarce water sources (Laurenson et al., 2012). However, wastewater may contain constituents of potential concern such as heavy metals, pathogens, and a high biological oxygen demand (Mosse et al., 2011). Furthermore, the salt content of these recycled waters, and the concentrations of specific salt ions (Na^+^, K^+^), is of paramount importance in relation to soil structure, vine performance, and berry and wine composition (Laurenson et al., 2012; Mosse et al., 2013; Netzer et al., 2014). In certain areas, such as the Mediterranean, water reuse can be considered as a cost-effective solution for agriculture since it reduces the need to develop new water resources and provides an adaptive solution to climate change along with an increase in the social and environmental value of water (Costa et al., 2016). Although wastewater use might mitigate drought stress, the short and mid-term detrimental effects of salt stress must be quantified, as pointed out by several authors (Laurenson et al., 2012; Costa et al., 2016).

Rising salinization of soil could pose a serious threat to grape growing because most irrigated vineyards, especially those deficit-irrigated, are at risk due to dissolved salts in irrigation water (Keller, 2010). The deleterious effects of salinity on plant growth are caused by an osmotic effect in which the increase in soluble salt concentration of the soil solution imposes an osmotic drought on the plant and a toxic effect in which the tissue concentrations of the micronutrient chloride and the beneficial element sodium increase to toxic levels (Marschner, 1986).

Salinity damage has been a concern for a long time in Australian vineyards (e.g., Hickinbotham and Williams, 1933; Walker et al., 2014); however, studies in other areas are scarce. Usually, tolerance of grapevines to salinity is measured by yield performance and by the capacity for salt exclusion, necessary to prevent salt damage to leaves and to minimize Cl^-^ and Na^+^ accumulation in grape juice and wine (Teakle and Tyerman, 2010). Nevertheless, the effects of salinity on berry or juice composition seem to depend on the combination of cultivar and rootstock and on the salt concentration in the irrigation water, as well as on its time of application over the growing season.

In a 6-year study on Colombard vines grafted onto Ramsey rootstock, Stevens et al. (2011) observed that saline irrigation applied at different stages over the growing cycle increased Na^+^ concentration in juice over the first four seasons but in the last two seasons this concentration only increased in some of the treatments. In contrast, Cl^-^ concentration in juice increased over the 6 years independently of the treatment. Interestingly, saline irrigation caused small variations in juice Brix, titratable acidity, pH, and malate concentration. Recently, Degaris et al. (2016) proved that ion partitioning in grapevines and thus Cl^-^, Na^+^, and K^+^ in berries and juice depends on the type of deficit irrigation applied in two red cultivars (Shiraz and Grenache). Partial root-zone drying reduced the concentration of these ions in the fruit of both cultivars when compared with a fully irrigated control and a deficit irrigated treatment.

The negative effects that salinity might provoke in grape composition can be reduced by the selection of a tolerant rootstock able to exclude salts. In a long-term trial, Walker et al. (2014) observed that Chardonnay and Shiraz vines showed a low yield and a high concentration of both chloride and sodium in grape juice (>500 mg/L) when they were own-rooted. However, Chardonnay on C5 and Shiraz on C7 rootstocks had the lowest concentration of grape juice chloride and sodium (<50 mg/L). Moreover, TSS in juice was significantly reduced when Chardonnay vines were own-rooted in comparison with those grafted on rootstocks. These authors noted also significant differences in pH and titratable acidity as a function of rootstock in both cultivars. Finally, Walker et al. (2014) highlighted the different responses between cultivars; Shiraz vines had been less affected by prolonged exposure to salinity when compared with Chardonnay vines. An interesting feature of this study was that significant correlations between juice chloride and sodium concentrations and those found in trunk wood were detected. Previously, Walker et al. (2000) had observed that, under salinity conditions, rootstock would influence color density and anthocyanin concentration in the berries, detecting significant differences among rootstocks for wine titratable acidity and wine score.

From the sensory point of view, salinity derived attributes (“brackish,” “seawater like,” “soapy”) are considered negative and had been correlated with high concentrations of Na, K, and Cl in wines (Mira de Orduña, 2010). In a study carried out on 4000 wines across 3 years, Kaufmann (1996) found a significant correlation between high chloride levels and arid producing regions. Average chloride levels of 0.69 mM across all European red and white wines analyzed contrasted with the 3.78 mM average for wines produced in the United States, Mexico, Argentina, and Australia.

## Summary, Conclusions, and Implications

Under the current scenario of global change, the constraints that water scarcity and salinity might induce on grape composition are becoming increasingly important worldwide. These stresses may endanger viticulture sustainability in the medium term by reducing yields and grape composition. Despite the huge amount of work aiming at assessing the effects of water status on vine yield and grape composition, no clear relationships could be established between Ψ_stem_ and berry size and composition. This is due to the large number of factors involved in grape composition development, indicating that water status might not be its main driver. The dataset analyzed in the current study proved that cultivar, timing of exposure to water restrictions and rootstock have a great influence on must and wine composition. Nevertheless, other factors, such as climate, leaf surface/yield ratio, training systems, amongst others, might interact with the ones that we focused on in the current study and should be taken into account for future research.

Water restrictions can be worsened by increasing salinity levels in soils and irrigation waters, especially in Mediterranean climates. Previous research proved that rootstocks possess different sensitivities to salinity levels in the soil and might reduce the concentration of saline ions in the fruit. Moreover, it seems that cultivars present also a different sensitivity to chloride and sodium.

## Author Contributions

Both JM-A and DI devised the structure and decided on the content of the paper, JM-A conducted the literature survey, and then JM-A wrote the manuscript. DI contributed to a general revision of the manuscript.

## Conflict of Interest Statement

The authors declare that the research was conducted in the absence of any commercial or financial relationships that could be construed as a potential conflict of interest.
