# Supplementary material for: Grape Composition under Abiotic Constrains: Water Stress and Salinity
Source: Front Plant Sci. 2017 May 30;8:851. doi: 10.3389/fpls.2017.00851 (PMC5447678; doi:10.3389/fpls.2017.00851)
Supplement: Supplementary file 1 [file Table_1.DOC]

**Supplementary Table 1.** Compilation of studies about irrigation strategies accounting for vine water status and determining agronomic parameters and quality of grape and wine in red and white grapevine varieties used in the meta-analysis.

| **Reference** | **Location** | **Cultivar** | **Measurements** |
| --- | --- | --- | --- |
| **Red grapevine varieties** | | | |
| Acevedo-Opazo et al. (2010) | Chile | Cabernet Sauvignon | A, M |
| Bindon et al. (2011) | South Africa | Merlot | A, M, W |
| Bucchetti et al. (2011) | Italy | Merlot | A, M |
| Chapman et al. (2005) | USA | Cabernet Sauvignon | A, M |
| Chaves et al. (2007) | Portugal | Castelão | A, M |
| De la Hera et al. (2007) | Spain | Monastrell | A, M |
| Deluc et al. (2009) | USA | Cabernet Sauvignon | A, M |
| Girona et al. (2006) | Spain | Pinot Noir | A, M |
| Gouveia et al. (2012) | Portugal | Touriga Nacional | A, M |
| Intrigliolo and Castel (2008) | Spain | Tempranillo | A, M, W |
| Intrigliolo and Castel (2009) | Spain | Tempranillo | A, M, W |
| Intrigliolo and Castel (2010) | Spain | Tempranillo | A, M, W |
| Intrigliolo and Castel (2011) | Spain | Tempranillo | A, M, W |
| Intrigliolo et al. (2012) | Spain | Tempranillo | A, M |
| Intrigliolo et al. (2016) | Spain | Cabernet Sauvignon | A, M |
| Junquera et al. (2012) | Spain | Cabernet Sauvignon | A, M |
| Keller et al. (2008) | USA | Cabernet Sauvignon | A, M |
| Kounduras et al. (2006) | Greece | Agiorgitiko | A, M, W |
| Lanari et al. (2014) | Italy | Sangiovese | A, M |
| Mendez-Costabel et al. (2014) | USA | Merlot | A, M, W |
| Munitz et al. (2017) | Israel | Merlot | A, M |
| Myburgh (2011a,b) | South Africa | Merlot | A, M |
| Romero et al. (2010) | Spain | Monastrell | A, M |
| Romero et al. (2013) | Spain | Monastrell | A, M, W |
| Salón et al. (2005) | Spain | Bobal | A, M |
| Santesteban et al. (2011) | Spain | Tempranillo | A, M |
| Shellie (2010) | USA | Merlot | A, M |
| Shellie (2014) | USA | Merlot | A, M |
| Shellie and Bowen (2014) | USA | Cabernet Sauvignon, Malbec | A, M |
| Trégoat et al. (2002) | France | Merlot | A, M |
| Williams (2012) | USA | Merlot | A, M |
| **White grapevine varieties** | | | |
| Balint and Reynolds (2013) | Canada | Sauvignon blanc | A, M, W |
| Balint and Reynolds (2017) | Canada | Chardonnay | A, M, W |
| Chaves et al. (2007) | Portugal | Muscat of Alexandria | A, M |
| Choné (2003) | France | Sauvignon blanc | A, M |
| Coombe and Monk (1979) | Australia | Riesling | A, M |
| Deluc et al. (2009) | USA | Chardonnay | A, M |
| Döring et al. (2015) | Germany | Riesling | A, M |
| dos Santos et al. (2007) | Portugal | Muscat of Alexandria | A, M |
| El-Ansary et al. (2005) | Japan | Muscat of Alexandria | A, M |
| El-Ansary and Okamoto (2007) | Japan | Muscat of Alexandria | A, M |
| Myburgh (2005, 2006) | South Africa | Sauvignon blanc | A, M |
| Greven et al. (2005) | New Zealand | Sauvignon blanc | A, M |
| Mirás-Avalos et al. (2016) | Spain | Albariño | A, M, W |
| Naor et al. (1993) | Israel | Sauvignon blanc | A, M |
| Reynolds et al. (2007) | Canada | Chardonnay | A, M, W |
| Savoi et al. (2016) | Italy | Sauvignon vert | A, M |
| Trigo-Córdoba et al. (2015) | Spain | Godello, Treixadura | A, M, W |

A = agronomic parameters; M = must composition; W = wine attributes
