# Supplementary material for: Grape Composition under Abiotic Constrains: Water Stress and Salinity
Source: Front Plant Sci. 2017 May 30;8:851. doi: 10.3389/fpls.2017.00851 (PMC5447678; doi:10.3389/fpls.2017.00851)
Supplement: Supplementary file 2 [file Table_2.DOC]

**Supplementary Table 2.** Variation in data distribution explained by the final model for each of the berry size, and berry and wine composition traits analyzed in the current study.

| **Variable** | **Variation explained by the model (%)** |
| --- | --- |
| **Red varieties** | |
| Berry weight | 96.4 |
| Total soluble solids | 48.9 |
| pH | 95.2 |
| Titratable acidity | 99.4 |
| Malic acid | 29.2 |
| Tartaric acid | 91.1 |
| Anthocyanins | 26.9 |
| Wine alcohol | 2.0 |
| Wine titratable acidity | 4.4 |
| Wine pH | 18.6 |
| Wine malic acid | 40.8 |
| Wine tartaric acid | 15.4 |
| Wine anthocyanins | 2.5 |
| Wine TPI | 2.0 |
| **White varieties** | |
| Berry weight | 74.3 |
| Total soluble solids | 34.4 |
| pH | 91.9 |
| Titratable acidity | 90.2 |
| Malic acid | 28.3 |
| Tartaric acid | 70.4 |
| Wine alcohol | 27.9 |
| Wine titratable acidity | 82.1 |
| Wine pH | 93.2 |
| Wine malic acid | 61.6 |
| Wine tartaric acid | 80.6 |

Model for red cultivars: Variable = Intercept + B_cultivar_ + B_timing_ + B_Ψσtem_

Model for white cultivars: Variable = Intercept + B_cultivar_ + B_Ψσtem_ + B_cultivar_ x Ψ_stem_
