# Supplementary material for: Grape Composition under Abiotic Constrains: Water Stress and Salinity
Source: Front Plant Sci. 2017 May 30;8:851. doi: 10.3389/fpls.2017.00851 (PMC5447678; doi:10.3389/fpls.2017.00851)
Supplement: Supplementary file 3 [file Table_3.DOC]

**Supplementary Table 3.** Pearson’s correlation coefficients (r) among different modalities of vine water status assessment and berry size and compositional traits for white cultivars. Significant correlations are shown in bold. Ψ_pd_ = pre-dawn leaf water potential, Ψ_l_ = midday leaf water potential, Ψ_stempre_ = pre-veraison midday stem water potential, Ψ_stempost_ = post-veraison midday stem water potential, Ψ_stem_ = midday stem water potential.

|  | | Ψ_pd_ | Ψ_l_ | Ψ_stempre_ | Ψ_stempost_ | Ψ_stem_ |
| --- | --- | --- | --- | --- | --- | --- |
| Berry weight | r | **0.222^*^** | -0.034 | **0.359^*^** | **0.328^*^** | **0.222^*^** |
|  | Significance | **0.027** | 0.771 | **0.027** | **0.044** | **0.027** |
|  | n | **99** | 77 | **38** | **38** | **99** |
| Total soluble solids | r | 0.024 | 0.068 | -0.236 | -0.066 | 0.024 |
|  | Significance | 0.789 | 0.527 | 0.128 | 0.673 | 0.789 |
|  | n | 122 | 90 | 43 | 43 | 122 |
| PH | r | -0.056 | 0.139 | -0.280 | -0.128 | -0.056 |
|  | Significance | 0.554 | 0.193 | 0.104 | 0.463 | 0.554 |
|  | n | 114 | 90 | 35 | 35 | 114 |
| Titratable acidity | r | **0.231^*^** | **0.314^**^** | 0.127 | **0.442^**^** | **0.231^*^** |
|  | Significance | **0.014** | **0.003** | 0.473 | **0.009** | **0.014** |
|  | n | **113** | **87** | 34 | **34** | **113** |
| Malic acid | r | 0.200 | 0.173 | 0.231 | 0.307 | 0.200 |
|  | Significance | 0.273 | 0.492 | 0.203 | 0.088 | 0.273 |
|  | n | 32 | 18 | 32 | 32 | 32 |
| Tartaric acid | r | -0.220 | **0.623^**^** | -0.135 | -0.119 | -0.220 |
|  | Significance | 0.243 | **0.006** | 0.477 | 0.531 | 0.243 |
|  | n | 30 | **18** | 30 | 30 | 30 |
| Wine alcohol | r | 0.119 | -0.115 | -0.288 | **-0.525^*^** | 0.119 |
|  | Significance | 0.437 | 0.454 | 0.246 | **0.025** | 0.437 |
|  | n | 45 | 45 | 18 | **18** | 45 |
| Wine titratable acidity | r | **0.372^*^** | 0.096 | 0.126 | 0.411 | **0.372^*^** |
|  | Significance | **.012** | 0.532 | 0.618 | 0.090 | **0.012** |
|  | n | **45** | 45 | 18 | 18 | **45** |
| Wine pH | r | **-0.507^**^** | -0.166 | -0.459 | **-0.678^**^** | **-0.507^**^** |
|  | Significance | **.000** | 0.275 | 0.055 | **0.002** | **0.000** |
|  | n | **45** | 45 | 18 | **18** | **45** |
| Wine malic acid | r | **-0.513^*^** | 0.094 | **-0.679^**^** | -0.445 | **-0.513^*^** |
|  | Significance | **.030** | 0.709 | **0.002** | 0.064 | **0.030** |
|  | n | **18** | 18 | **18** | 18 | **18** |
| Wine tartaric acid | r | **0.650^**^** | 0.224 | **0.487^*^** | **0.631^**^** | **0.650^**^** |
|  | Significance | **.004** | 0.371 | **0.040** | **.005** | **.004** |
|  | n | **18** | 18 | **18** | **18** | **18** |
