# Supplementary material for: Grape Composition under Abiotic Constrains: Water Stress and Salinity
Source: Front Plant Sci. 2017 May 30;8:851. doi: 10.3389/fpls.2017.00851 (PMC5447678; doi:10.3389/fpls.2017.00851)

Supplementary Figure 1. Relationships between midday stem water potential over the growing season and several berry traits for red cultivars: (A) fresh weight, (B) total soluble solids, (C) titratable acidity, and (D) anthocyanins concentration.

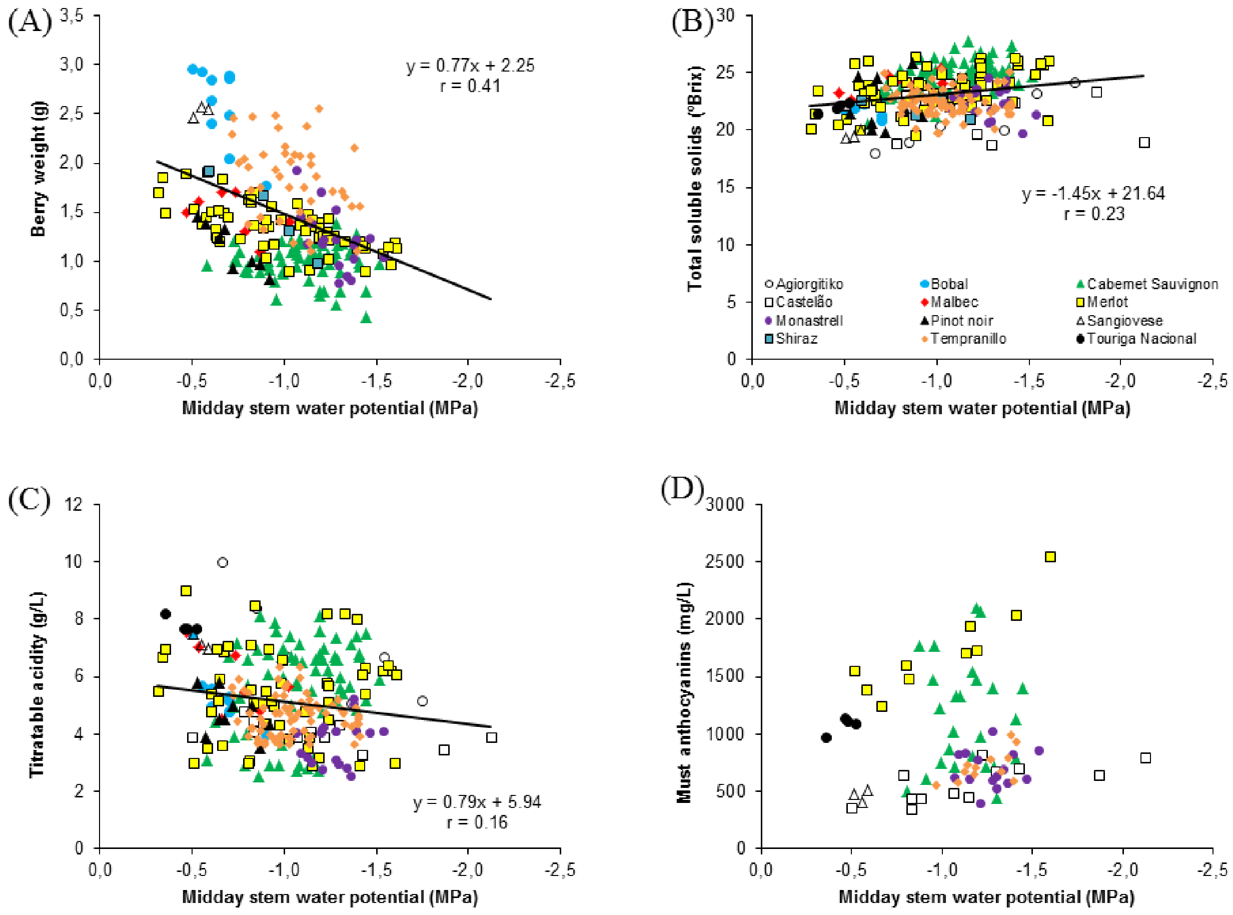

Supplement: Supplementary file 4 [file Image_1.PDF]
